# Induced Pluripotent Stem Cells: Problems and Advantages when Applying them in Regenerative Medicine

**Published:** 2010-07

**Authors:** S.P. Medvedev, A.I. Shevchenko, S.M. Zakian

**Affiliations:** Institute of Cytology and Genetics, Siberian Branch, Russian Academy of Sciences; Research Center of Clinical and Experimental Medicine, Siberian Branch, Russian Academy of Medical Sciences

**Keywords:** induced pluripotent stem cells, directed stem cell differentiation, cell replacement therapy

## Abstract

Induced pluripotent stem cells (iPSCs) are a new type of pluripotent cells that can be obtained by reprogramming animal and human differentiated cells. In this review, issues related to the nature of iPSCs are discussed and different methods of iPSC production are described. We particularly focused on methods of iPSC production without the genetic modification of the cell genome and with means for increasing the iPSC production efficiency. The possibility and issues related to the safety of iPSC use in cell replacement therapy of human diseases and a study of new medicines are considered.

## INDUCED PLURIPOTENCY

Pluripotent stem cells are a unique model for studying a variety of processes that occur in the early development of mammals and a promising tool in cell therapy of human diseases. The unique nature of these cells lies in their capability, when cultured, for unlimited self–renewal and reproduction of all adult cell types in the course of their differentiation [1]. Pluripotency is supported by a complex system of signaling molecules and gene network that is specific for pluripotent cells. The pivotal position in the hierarchy of genes implicated in the maintenance of pluripotency is occupied by * Oct4, Sox2 * , and * Nanog * genes encoding transcription factors [2, 3]. The mutual effect of outer signaling molecules and inner factors leads to the formation of a specific expression pattern, as well as to the epigenome state characteristic of stem cells. Both spontaneous and directed differentiations are associated with changes in the expression pattern and massive epigenetic transformations, leading to transcriptome and epigenome adjustment to a distinct cell type.

Until recently, embryonic stem cells (ESCs) were the only well–studied source of pluripotent stem cells. ESCs are obtained from either the inner cell mass or epiblast of blastocysts [4–6]. A series of protocols has been developed for the preparation of various cell derivatives from human ESCs. However, there are constraints for ESC use in cell replacement therapy. The first constraint is the immune incompatibility between the donor cells and the recipient, which can result in the rejection of transplanted cells. The second constraint is ethical, because the embryo dies during the isolation of ESCs. The first problem can be solved by the somatic cell nuclear transfer into the egg cell and then obtaining the embryo and ESCs. The nuclear transfer leads to genome reprogramming, in which ovarian cytoplasmic factors are implicated. This way of preparing pluripotent cells from certain individuals was called therapeutic cloning. However, this method is technology–intensive, and the reprogramming yield is very low. Moreover, this approach encounters the above–mentioned ethic problem that, in this case, is associated with the generation of many human ovarian cells [7].

In 2006, the preparation of pluripotent cells by the ectopic expression of four genes – *Oct4 * , * Sox2 * , * Klf4 * , and *c–Myc * – in both embryonic and adult murine fibroblasts was first reported [8]. The pluripotent cells derived from somatic ones were called induced pluripotent stem cells (iPSCs). Using this set of factors (Oct4, Sox2, Klf4, and c–Myc), iPSCs were prepared later from various differentiated mouse [9–14] and human [15–17] cell types. Human iPSCs were obtained with a somewhat altered gene set: * Oct4 * , * Sox2 * , *Nanog * , and * Lin28 * [18]. Induced PSCs closely resemble ESCs in a broad spectrum of features. They possess similar morphologies and growth manners and are equally sensitive to growth factors and signaling molecules. Like ESCs, iPSCs can differentiate * in vitro * into derivatives of all three primary germ layers (ectoderm, mesoderm, and endoderm) and form teratomas following their subcutaneous injection into immunodeficient mice. Murine iPSCs injected into blastocysts are normally included in the development to yield animals with a high degree of chimerism. Moreover, murine iPSCs, when injected into tetraploid blastocycts, can develop into a whole organism [19, 20]. Thus, an excellent method that allows the preparation of pluripotent stem cells from various somatic cell types while bypassing ethical problems has been uncovered by researchers.

## The problem of ipsc production efficiency and application safety in cell replacement therapy

In the first works on murine and human iPSC production, either retro– or lentiviral vectors were used for the delivery of * Oct4 * , * Sox2 * , *Klf4 * , and * c–Myc * genes into somatic cells. The efficiency of transduction with retroviruses is high enough, although it is not the same for different cell types. Retroviral integration into the host genome requires a comparatively high division rate, which is characteristic of the relatively narrow spectrum of cultured cells. Moreover, the transcription of retroviral construct under the control of a promoter localized in 5’LTR (long terminal repeat) is terminated when the somatic cell transform switches to the pluripotent state [21]. This feature makes retroviruses attractive in iPSC production. Nevertheless, retroviruses possess some properties that make iPSCs that are produced using them improper for cell therapy of human diseases. First, retroviral DNA is integrated into the host cell genome. The integration occurs randomly; i.e., there are no specific sequences or apparent logic for retroviral integration. The copy number of the exogenous retroviral DNA that is integrated into a genome may vary to a great extent [15]. Retroviruses being integrated into the cell genome can introduce promoter elements and polyadenylation signals; they can also interpose coding sequences, thus affecting transcription. Second, since the transcription level of exogenous * Oct4 * , * Sox2 * , *Klf4 * , and * c–Myc * in the retroviral construct decreases with cell transition into the pluripotent state, this can result in a decrease in the efficiency of the stable iPSC line production, because the switch from the exogenous expression of pluripotency genes to their endogenous expression may not occur. Third, some studies show that the transcription of transgenes can resume in the cells derived from iPSCs [22]. The high probability that the ectopic * Oct4 * , * Sox2 * , * Klf4 * , and *c–Myc * gene expression will resume makes it impossible to apply iPSCs produced with the use of retroviruses in clinical trials; moreover, these iPSCs are hardly applicable even for fundamental studies on reprogramming and pluripotency principles. Lentiviruses used for iPSC production can also be integrated into the genome and maintain their transcriptional activity in pluripotent cells. One way to avoid this situation is to use promoters controlled by exogenous substances added to the culture medium, such as tetracycline and doxycycline, which allows the transgene transcription to be regulated. iPSCs are already being produced using such systems [23].

Another serious problem is the gene set itself that is used for the induction of pluripotency [22]. The ectopic transcription of * Oct4* , * Sox2 * , * Klf4 * , and * c–Myc* can lead to neoplastic development from cells derived from iPSCs, because the expression of * Oct4 * , * Sox2 * , * Klf4,* and * c–Myc * genes is associated with the development of multiple tumors known in oncogenetics [22, 24]. In particular, the overexpression of * Oct4* causes murine epithelial cell dysplasia [25], the aberrant expression of * Sox2 * causes the development of serrated polyps and mucinous colon carcinomas [26], breast tumors are characterized by elevated expression of * Klf4 * [27] * , * and the improper expression of * c–Myc* is observed in 70% of human cancers [28]. Tumor development is oberved in ~50% of murine chimeras obtained through the injection of retroviral iPSCs into blastocysts, which is very likely associated with the reactivation of exogenous * c–Myc * [29, 30].

Several possible strategies exist for resolving the above-mentioned problems:

- The search for a less carcinogenic gene set that is necessary and sufficient for reprogramming;
- The minimization of the number of genes required for reprogramming and searching for the nongenetic factors facilitating it;
- The search for systems allowing the elimination of the exogenous DNA from the host cell genome after the reprogramming;
- The development of delivery protocols for nonintegrated genetic constructs;
- The search for ways to reprogram somatic cells using recombinant proteins.

## Possible gene substituents for * c-Myc * and * Klf4 * in IPSC preparation

The ectopic expression of * c–Myc * and * Klf4 * genes is the most dangerous because of the high probability that malignant tumors will develop [22]. Hence the necessity to find other genes that could substitute * c–Myc * and * Klf4 * in iPSC production. It has been reported that these genes can be successfully substituted by * Nanog* and * Lin28 * for reprogramming human somatic cells [18;] . iPSCs were prepared from murine embryonic fibroblasts by the overexpression of * Oct4 * and * Sox2 * , as well as the *Esrrb * gene encoding the murine orphan nuclear receptor beta. It has already been shown that * Esrrb * , which acts as a transcription activator of *Oct4 * , * Sox2 * , and * Nanog * , is necessary for the self–renewal and maintenance of the pluripotency of murine ESCs. Moreover, * Esrrb* can exert a positive control over * Klf4 * . Thus, the genes causing elevated carcinogenicity of both iPSCs and their derivatives can be successfully replaced with less dangerous ones [31].

## Means for increasing the production efficiency of ipscs

**The Most Effectively Reprogrammed Cell Lines **. Murine and human iPSCs can be obtained from fibroblasts using the factors Oct4, Sox2, and Klf4, but without * c–Myc * . However, in this case, reprogramming decelerates and an essential shortcoming of stable iPSC clones is observed [32, 33]. The reduction of a number of necessary factors without any decrease in efficiency is possible when iPSCs are produced from murine and human neural stem cells (NSCs) [12, 34, 35]. For instance, iPSCs were produced from NSCs isolated from adult murine brain using two factors, Oct4 and Klf4, as well as even Oct4 by itself [12, 34]. Later, human iPSCs were produced by the reprogramming of fetal NSCs transduced with a retroviral vector only carrying * Oct4* [35] . It is most likely that the irrelevance of Sox2, Klf4, and c–Myc is due to the high endogenous expression level of these genes in NSCs. 

 Successful reprogramming was also achieved in experiments with other cell lines, in particular, melanocytes of neuroectodermal genesis [36]. Both murine and human melanocytes are characterized by a considerable expression level of the * Sox2 * gene, especially at early passages. iPSCs from murine and human melanocytes were produced without the use of Sox2 or c–Myc. However, the yield of iPSC clones produced from murine melanocytes was lower (0.03% without Sox2 and 0.02% without c–Myc) in comparison with that achieved when all four factors were applied to melanocytes (0.19%) and fibroblasts (0.056%). A decreased efficiency without Sox2 or c–Myc was observed in human melanocyte reprogramming (0.05% with all four factors and 0.01% without either * Sox2 * or * c–Myc* ). All attempts to obtain stable iPSC clones in the absence of both Sox2 and c–Myc were unsuccessful [36]. Thus, the minimization of the number of factors required for iPSC preparation can be achieved by choosing the proper somatic cell type that most effectively undergoes reprogramming under the action of fewer factors, for example, due to the endogenous expression of “pluripotency genes.” However, if human iPSCs are necessary, these somatic cells should be easily accessible and well–cultured and their method of isolation should be as noninvasive as possible.

One of these cell types can be adipose stem cells (ASCs). This is a heterogeneous group of multipotent cells which can be relatively easily isolated in large amounts from adipose tissue following liposuction. Human iPSCs were successfully produced from ASCs with a twofold reprogramming rate and 20–fold efficiency (0.2%), exceeding those of fibroblasts [37].

However, more accessible resources for the effective production of human iPSCs are keratinocytes. When compared with fibroblasts, human iPSC production from keratinocytes demonstrated a 100–fold greater efficiency and a twofold higher reprogramming rate [38].

It has recently been found that the reprogramming of murine papillary dermal fibroblasts (PDFs) into iPSCs can be highly effective with the overexpression of only two genes, * Oct4 * and * Klf4 * , inserted into retroviral vectors [39;]. PDFs are specialized cells of mesodermal genesis surrounding the stem cells of hair follicles . One characteristic feature of these cells is the endogenous expression of *Sox2 * , * Klf4 * , and * c–Myc * genes, as well as the gene–encoding alkaline phosphatase, one of the murine and human ESC markers. PDFs can be easily separated from other cell types by FACS (fluorescence–activated cell sorting) using life staining with antibodies against the surface antigens characteristic of one or another cell type. The PDF reprogramming efficiency with the use of four factors (Oct4, Sox2, Klf4, and c–Myc) retroviral vectors is 1.38%, which is 1,000–fold higher than the skin fibroblast reprogramming efficiency in the same system. Reprogramming PDFs with two factors, * Oct4* and * Klf4 * , yields 0.024%, which is comparable to the efficiency of skin fibroblast reprogramming using all four factors. The efficiency of PDF reprogramming is comparable with that of NSCs, but PDF isolation is steady and far less invasive [39]. It seems likely that human PDF lines are also usable, and this cell type may appear to be one of the most promising for human iPSC production in terms of pharmacological studies and cell replacement therapy. The use of such cell types undergoing more effective reprogramming, together with methods providing the delivery of “pluripotency genes” without the integration of foreign DNA into the host genome and chemical compounds increasing the reprogramming efficiency and substituting some factors required for reprogramming, is particularly relevant.

**Chemical Compounds Increasing Cell Reprogramming Efficiency**. As was noted above, the minimization of the factors used for reprogramming decreases the efficiency of iPSC production. Nonetheless, several recent studies have shown that the use of genetic mechanisms, namely, the initiation of ectopic gene expression, can be substituted by chemical compounds, most of them operating at the epigenetic level. For instance, BIX–01294 inhibiting histone methyltransferase G9a allows murine fibroblast reprogramming using only two factors, Oct4 and Klf4, with a fivefold increased yield of iPSC clones in comparison with the control experiment without BIX–01294 [40]. BIX–01294 taken in combination with another compound can increase the reprogramming efficiency even more. In particular, BIX–01294 plus BayK8644 elevated the yield of iPCSs 15 times, and BIX–01294 plus RG108 elevated it 30 times when only two reprogramming factors, Oct4 and Klf4, were used. RG108 is an inhibitor of DNA methyltransferases, and its role in reprogramming is apparently in initiating the more rapid and effective demethylation of promoters of pluripotent cell–specific genes, whereas BayK8644 is an antagonist of L–type calcium channels, and its role in reprogramming is not understood very well [40]. However, more considerable results were obtained in reprogramming murine NSCs. The use of BIX–01294 allowed a 1.5–fold increase in iPSC production efficiency with two factors, Oct4 and Klf4, in comparison with reprogramming with all four factors. Moreover, BIX–01294 can even substitute Oct4 in the reprogramming of NSCs, although the yield is very low [41]. Valproic (2–propylvaleric) acid inhibiting histone deacetylases can also substitute c–Myc in reprogramming murine and human fibroblasts. Valproic acid (VPA) increases the reprogramming efficiency of murine fibroblasts 50 times, and human fibroblasts increases it 10–20 times when three factors are used [42, 43]. Other deacetylase inhibitors, such as TSA (trichostatin A) and SAHA (suberoylanilide hyroxamic acid), also increase the reprogramming efficiency. TSA increases the murine fibroblast reprogramming efficiency 15 times, and SAHA doubles it when all four factors are used [42]. Besides epigenetic regulators, the substances inhibiting the protein components of signaling pathways implicated in the differentiation of pluripotent cells are also applicable in the substitution of reprogramming factors. In particular, inhibitors of MEK and GSK3 kinases (PD0325901 and CHIR99021, respectively) benefit the establishment of the complete and stable pluripotency of iPSCs produced from murine NSCs using two factors, Oct4 and Klf4 [41, 44].

It has recently been shown that antioxidants can considerably increase the efficiency of somatic cell reprogramming. Ascorbic acid (vitamin C) can essentially influence the efficiency of iPSC production from various murine and human somatic cell types [45]. The transduction of murine embryonic fibroblasts (mEFs) with retroviruses carrying the * Oct4 * , * Sox2 * , and * Klf4* genes results in a significant increase in the production level of reactive oxygen species (ROS) compared with that of both control and Efs tranduced with * Oct4 * , *Sox2 * , * c–Myc * , and * Klf4 * . In turn, the increase in the ROS level causes accelerated aging and apoptosis of the cell, which should influence the efficiency of cell reprogramming. By testing several substances possessing antioxidant activity such as vitamin B1, sodium selenite, reduced glutathione, and ascorbic acid, the authors have found that combining these substances increases the yield of GFP–positive cells in EF reprogramming (the * Gfp * gene was under the control of the * Oct4 * gene promoter). The use of individual substances has shown that only ascorbate possesses a pronounced capability to increase the level of GFP–positive cells, although other substances keep their ROS–decreasing ability. In all likelihood, this feature of ascorbates is not directly associated with its antioxidant activity [45]. The score of GFP–positive iPSC colonies expressing an alkaline phosphatase has shown that the efficiency of iPSC production from mEFs with three factors (Oct4, Sox2, and Klf4) can reach 3.8% in the presence of ascorbate. When all four factors (Oct4, Sox2, Klf4, and c–Myc) are used together with ascorbate, the efficiency of iPSC production may reach 8.75%. A similar increase in the iPSC yield was also observed in the reprogramming of murine breast fibroblasts; i.e., the effect of vitamin C is not limited by one cell type. Moreover, the effect of vitamin C on the reprogramming efficiency is more profound than that of the deacetylase inhibitor valproic (2–propylvaleric) acid. The mutual effect of ascorbate and valproate is additive; i.e., these substances have different action mechanisms. Moreover, vitamin C facilitates the transition from pre–iPSCs to stable pluripotent cells. This feature is akin to the effects of PD0325901 and CHIR99021, which are inhibitors of MEK and GSK3 kinases, respectively. This effect of vitamin C expands to human cells as well [45]. Following the transduction of human fibroblasts with retroviruses carrying * Oct4 * , * Sox2 * , *Klf4 * , and * c–Myc * and treatment with ascorbate, the authors prepared iPSCs with efficiencies reaching 6.2%. The reprogramming efficiency of ASCs under the same conditions reached 7.06%. The mechanism of the effect that vitamin C has on the reprogramming efficiency is not known in detail. Nevertheless, the acceleration of cell proliferation was observed at the transitional stage of reprogramming. The levels of the p53 and p21 proteins decreased in cells treated with ascorbate, whereas the DNA repair machinery worked properly [45]. It is interesting that an essential decrease in the efficiency of iPSC production has been shown under the action of processes initiated by p53 and p21 [46–50].

## Methods for ipsc production without modification of the cell genome

As was mentioned above, for murine and human iPSC production, both retro– and lentiviruses were initially used as delivery vectors for the genes required for cell reprogramming. The main drawback of this method is the uncontrolled integration of viral DNA into the host cell’s genome. Several research groups have introduced methods for delivering “pluripotency genes” into the recipient cell which either do not integrate allogenic DNA into the host genome or eliminate exogenous genetic constructs from the genome.

***Cre–loxP–* Mediated Recombination**. To prepare iPSCs from patients with Parkinson’s disease, lentiviruses were used, the proviruses of which can be removed from the genome by * Cre* –recombinase. To do this, the * loxP * –site was introduced into the lentiviral 3’LTR–regions containing separate reprogramming genes under the control of the doxycycline–inducible promoter. During viral replication, *loxP * was duplicated in the 5’LTR of the vector. As a result, the provirus integrated into the genome was flanked with two * loxP* –sites. The inserts were eliminated using the temporary transfection of iPSCs with a vector expressing * Cre * –recombinase [51].

In another study, murine iPSCs were produced using a plasmid carrying the *Oct4 * , * Sox2 * , * Klf4I, * and *c–Myc * genes in the same reading frame in which individual cDNAs were separated by sequences encoding 2А peptides, and practically the whole construct was flanked with *loxP * –sites [52]. The use of this vector allowed a notable decrease in the number of exogenous DNA inserts in the host cell’s genome and, hence, the simplification of their following excision [52]. It has been shown using lentiviruses carrying similar polycistronic constructs that one copy of transgene providing a high expression level of the exogenous factors Oct4, Sox2, Klf4, and c–Myc is sufficient for the reprogramming of differentiated cells into the pluripotent state [53, 54].

The drawback of the * Cre–loxP * –system is the incomplete excision of integrated sequences; at least the * loxP * –site remains in the genome, so the risk of insertion mutations remains.

**Plasmid Vectors **. The application of lentiviruses and plasmids carrying the *loxP * –sites required for the elimination of transgene constructs modifies, although insignificantly, the host cell’s genome. One way to avoid this is to use vector systems that generally do not provide for the integration of the whole vector or parts of it into the cell’s genome. One such system providing a temporary transfection with polycistronic plasmid vectors was used for iPSC production from mEFs [29]. A polycistronic plasmid carrying the * Oct4* , * Sox2 * , and * Klf4 * gene cDNAs, as well as a plasmid expressing * c–Myc * , was transfected into mEFs one, three, five, and seven days after their primary seeding. Fibroblasts were passaged on the ninth day, and the iPSC colonies were selected on the 25th day. Seven out of ten experiments succeeded in producing GFP–positive colonies (the * Gfp * gene was under the control of the * Nanog * gene promoter). The iPSCs that were obtained were similar in their features to murine ESCs and did not contain inserts of the used DNA constructs in their genomes. Therefore, it was shown that wholesome murine iPSCs that do not carry transgenes can be reproducibly produced, and that the temporary overexpression of * Oct4 * , * Sox2 * , * Klf4* , and * c–Myc * is sufficient for reprogramming. The main drawback of this method is its low yield. In ten experiments the yield varied from 1 to 29 iPSC colonies per ten million fibroblasts, whereas up to 1,000 colonies per ten millions were obtained in the same study using retroviral constructs [29].

**Episomal Vectors **. Human iPSCs were successfully produced from skin fibroblasts using single transfection with polycistronic episomal constructs carrying various combinations of * Oct4 * , * Sox2 * , * Nanog* , * Klf4 * , * c–Myc * , * Lin28* , and * SV40LT * genes. These constructs were designed on the basis of the oriP/EBNA1 (Epstein–Barr nuclear antigen–1) vector [55]. The oriP/EBNA1 vector contains the IRES2 linker sequence allowing the expression of several individual cDNAs (encoding the genes required for successful reprogramming in this case) into one polycistronic mRNA from which several proteins are translated. The oriP/EBNA1 vector is also characterized by low–copy representation in the cells of primates and can be replicated once per cell cycle (hence, it is not rapidly eliminated, the way common plasmids are). Under nonselective conditions, the plasmid is eliminated at a rate of about 5% per cell cycle [56]. In this work, the broad spectrum of the reprogramming factor combinations was tested, resulting in the best reprogramming efficiency with cotransfection with three episomes containing the following gene sets: * Oct4* *+* *Sox2* *+* *Nanog* *+* *Klf4 * , *Oct4* *+* *Sox2 * + * SV40LT* + * Klf4 * , and * c–Myc* *+* *Lin28 * . * SV40LT * ( * SV40 large T gene * ) neutralizes the possible toxic effect of * с–Мус* overexpression [57]. The authors have shown that wholesome iPSCs possessing all features of pluripotent cells can be produced following the temporary expression of a certain gene combination in human somatic cells without the integration of episomal DNA into the genome. However, as in the case when plasmid vectors are being used, this way of reprogramming is characterized by low efficiency. In separate experiments the authors obtained from 3 to 6 stable iPSC colonies per 10^6^ transfected fibroblasts [55]. Despite the fact that skin fibroblasts are well–cultured and accessible, the search for other cell types which are relatively better cultured and more effectively subject themselves to reprogramming through this method is very likely required. Another drawback of the given system is that this type of episome is unequally maintained in different cell types.

***PiggyBac*–Transposition **. One promising system used for iPSC production without any modification of the host genome is based on DNA transposons. So–called * PiggyBac * –transposons containing 2А–linkered reprogramming genes localized between the 5’– and 3’–terminal repeats were used for iPSC production from fibroblasts. The integration of the given constructs into the genome occurs due to mutual transfection with a plasmid encoding transposase. Following reprogramming due to the temporary expression of transposase, the elimination of inserts from the genome took place [58, 59]. One advantage of the *PiggyBac * system on * Cre–loxP * is that the exogenous DNA is completely removed [60].

However, despite the relatively high efficiency of exogenous DNA excision from the genome by *PiggyBac * –transposition, the removal of a large number of transposon copies is hardly achievable.

**Nonintegrating Viral Vectors **. Murine iPSCs were successfully produced from hepatocytes and fibroblasts using four adenoviral vectors nonintegrating into the genome and carrying the * Oct4 * , * Sox2 * , * Klf4* , and * c–Myc * genes. An analysis of the obtained iPSCs has shown that they are similar to murine ESCs in their properties (teratoma formation, gene promoter DNA methylation, and the expression of pluripotent markers), but they do not carry insertions of viral DNA in their genomes [61]. Later, human fibroblast–derived iPSCs were produced using this method [62].

The authors of this paper cited the postulate that the use of adenoviral vectors allows the production of iPSCs, which are suitable for use without the risk of viral or oncogenic activity. Its very low yield (0.0001–0.001%), the deceleration of reprogramming, and the probability of tetraploid cell formation are the drawbacks of the method. Not all cell types are equally sensitive to transduction with adenoviruses.

Another method of gene delivery based on viral vectors was recently employed for the production of human iPSCs. The sendai–virus (SeV)–based vector was used in this case [63]. SeV is a single–stranded RNA virus which does not modify the genome of recipient cells; it seems to be a good vector for the expression of reprogramming factors. Vectors containing either all “pluripotency factors” or three of them (without * с–Мус* ) were used for reprogramming the human fibroblast. The construct based on SeV is eliminated later in the course of cell proliferation. It is possible to remove cells with the integrated provirus via negative selection against the surface HN antigen exposed on the infected cells. The authors postulate that reprogramming technology based on SeV will enable the production of clinically applicable human iPSCs [63].

**Cell Transduction with Recombinant Proteins **. Although the methods for iPSC production without gene modification of the cell’s genome (adenoviral vectors, plasmid gene transfer, etc.) are elaborated, the theoretical possibility for exogenous DNA integration into the host cell’s genome still exists. The mutagenic potential of the substances used presently for enhancing iPSC production efficiency has not been studied in detail. Fully checking iPSC genomes for exogenous DNA inserts and other mutations is a difficult task, which becomes impossible to solve in bulk culturing of multiple lines. The use of protein factors delivered into a differentiated cell instead of exogenous DNA may solve this problem. Two reports have been published to date in which murine and human iPSCs were produced using the recombinant Oct4, Sox2, Klf4, and c–Myc proteins [64, 65] . T he method used to deliver the protein into the cell is based on the ability of peptides enriched with basic residues (such as arginine and lysine) to penetrate the cell’s membrane. Murine iPSCs were produced using the recombinant Oct4, Sox2, Klf4, and c–Myc proteins containing eleven C–terminal arginine residues and expressed in * E. coli* . The authors succeeded in producing murine iPSCs during four rounds of protein transduction into embryonic fibroblasts [65]. However, iPSCs were only produced when the cells were additionally treated with 2–propylvalerate (the deacetylase inhibitor). The same principle was used for the production of human iPSCs, but protein expression was carried out in human HEK293 cells, and the proteins were expressed with a fragment of nine arginins at the protein C–end. Researchers have succeeded in producing human iPSCs after six transduction rounds without any additional treatment [64]. The efficiency of producing human iPSC in this way was 0.001%, which is one order lower than the reprogramming efficiency with retroviruses. Despite some drawbacks, this method is very promising for the production of patient–specific iPSCs.

## Induced pluripotent stem cells as a model for pathogenesis studies and a source of cell replacement therapy

The first lines of human pluripotent ESCs were produced in 1998 [6]. In line with the obvious fundamental importance of embryonic stem cell studies with regard to the multiple processes taking place in early embryogenesis, much of the interest of investigators is associated with the possibility of using ESCs and their derivatives as models for the pathogenesis of human diseases, new drugs testing, and cell replacement therapy. Substantial progress is being achieved in studies on directed human ESC differentiation and the possibility of using them to correct degenerative disorders. Functional cell types, such as motor dopaminergic neurons, cardiomyocytes, and hematopoietic cell progenitors, can be produced as a result of ESC differentiation. These cell derivatives, judging from their biochemical and physiological properties, are potentially applicable for the therapy of cardiovascular disorders, nervous system diseases, and human hematological disorders [66]. Moreover, derivatives produced from ESCs have been successfully used for treating diseases modeled on animals. Therefore, blood–cell progenitors produced from ESCs were successfully used for correcting immune deficiency in mice. Visual functions were restored in blind mice using photoreceptors produced from human ESCs, and the normal functioning of the nervous system was restored in rats modeling Parkinson’s disease using the dopaminergic neurons produced from human ESCs [67–70]. Despite obvious success, the full–scale application of ESCs in therapy and the modeling of disorders still carry difficulties, because of the necessity to create ESC banks corresponding to all HLA–haplotypes, which is practically unrealistic and hindered by technical and ethical problems.

**Table 1:** Functional categories of M. tuberculosis genes with changed expression level during transition to the NC state

Disease	Causative factor	Reprogrammed cell type	Means of reprogramming	Ref. No
Adenosine deaminase deficiency	Replacement of GGG with AGG in exon 7 resulting in G216R substitution or deletion of GAAGA in exon 10 of the *ADA* (*adenosine deaminase*) gene	Skin fibroblasts, karyotype 46,XY	Transduction with retroviruses carrying the *OCT4*, *SOX2*, *KLF4*, and *c-MYC* cDNAs	[91]
Type 3 Gaucher’s disease	Replacement of AAC with AGC in exon 9 or insertion of G at position 84 of the GBA (*β-acid glucosidase*) gene cDNA	Skin fibroblasts, karyotype 46,XY	Transduction with retroviruses carrying the *OCT4*, *SOX2*, *KLF4*, and *c-MYC* cDNAs	[91]
Duchenne muscular dystrophy	Deletion of exons 45-52 of the DMD (*dystrophin*) gene	Skin fibroblasts, karyotype 46,XY	Transduction with retroviruses carrying the *OCT4*, *SOX2*, *KLF4*, and *c-MYC* cDNAs	[91]
Becker muscular dystrophy	Unidentified mutation in the *DMD* gene	Skin fibroblasts, karyotype 46,XY	Transduction with retroviruses carrying the *OCT4*, *SOX2*, *KLF4*, and *c-MYC* cDNAs	[91]
Down syndrome	Trisomy of chromosome 21	Skin fibroblasts, karyotype 47,XY,+21	Transduction with retroviruses carrying the *OCT4*, *SOX2*, *KLF4*, and *c-MYC* cDNAs	[91]
Parkinson’s disease	Multifactorial disease	Skin fibroblasts, karyotype 46,XY	Transduction with retroviruses carrying the *OCT4*, *SOX2*, *KLF4*, and *c-MYC* cDNAs	[91]
		Fibroblasts; the age of the patient at the moment of biopsy was 53–85 years, karyotypes: 46,XY (six lines) and 46,XX (one line)	Transduction with lentiviruses carrying the *OCT4*, *SOX2*, and *KLF4* or *OCT4*, *SOX2*, *KLF4* and *c-MYC* genes. Viral LTRs contained LoxP sites required for the excision of the exogenous construct from the cell genome	[51]
Diabetes mellitus type 1 (juvenile diabetes)	Multifactorial disease	Skin fibroblasts, karyotype 46,XX	Transduction with retroviruses carrying the *OCT4*, *SOX2*, *KLF4*, and *c-MYC* cDNAs	[91]
Shwachman–Bodian–Diamond syndrome	Point mutations in the SBDS (*Shwachman–Bodian–Diamond Syndrome*) gene	Bone marrow mesenchymal cells, karyotype 46,XY	Transduction with retroviruses carrying the *OCT4*, *SOX2*, *KLF4*, and *c-MYC* cDNAs	[91]
Huntington’s disease	CAG repeat expansion in the Huntington gene from normal 11–34 copies to 37–100 and more	Skin fibroblasts, karyotype 46,XX	Transduction with retroviruses carrying the *OCT4*, *SOX2*, *KLF4*, and *c-MYC* cDNAs	[91]
Lesch–Nyhan syndrome	Mutations in the HPRT (hypoxanthine-guanine phosphoribosyltransferase) gene	Skin fibroblasts, karyotype 46,XX	Transduction with retroviruses carrying the *OCT4*, *SOX2*, *KLF4*, and *c-MYC* cDNAs. One line was produced by transduction with doxycyclin-controlled lentiviral vectors carrying the OCT4, SOX2, KLF4, c-MYC, and NANOG cDNAs	[91]
		Fibroblasts, karyotype 46,XX	Transduction with lentiviruses carrying the *OCT4*, *SOX2*, and *KLF4* genes. Viral LTRs contained *LoxP* sites required for the excision of exogenous construct from cell genome	[51]
Dyskeratosis congenita (Zinsser-Engman-Cole syndrome)	Mutations in the DKC1 (*Dyskeratosis congenita*) gene	Fibroblasts, karyotype 46,XX	Transduction with lentiviruses carrying the *OCT4*, *SOX2*, and *KLF4* genes	[51]
Spinal muscular atrophy	Mutations in the SMN1 (*Survival Motor Neuron 1*) gene resulting in a decreased level of the *SMN* protein	Skin fibroblasts, karyotype 46,XY	Transduction with lentiviruses carrying the *OCT4*, *SOX2*, *NANOG*, and *LIN28* cDNAs	[89]
Familial dysautonomia	Mutation in the IKBKAP (*inhibitor of kappa light polypeptide gene enhancer in B-cells; IkB kinase complex associated protein*) gene resulted in shift splicing that generates a transcript lacking exon 20	Lung and skin fibroblasts, karyotypes 46,XX and 46,XY	Transduction with lentiviruses carrying the *OCT4*, *SOX2*, *KLF4*, and *c-MYC* cDNAs	[90]
β-Thalassemia	Mutations in the HBB (*haemoglobin beta*) gene	Skin fibroblasts, karyotype 46,XY	Transduction with retroviruses carrying the *OCT4*, *SOX2*, *KLF4*, and *c-MYC* cDNAs	[92]
Diabetes mellitus type 1 (juvenile diabetes)	Multifactorial disease	Skin fibroblasts, karyotype 46,XY	Transduction with retroviruses carrying the *OCT4*, *SOX2*, and *KLF4* cDNAs	[93]
Amyotrophic lateral sclerosis	L144F substitution in superoxide dismutase encoded by the dominant allele of the SOD1 (*Superoxide dismutase 1*) gene; this mutation is associated with slow disease progression	Skin fibroblasts, karyotype 46,XX	Transduction with retroviruses carrying the *OCT4*, *SOX2*, *KLF4*, and *c-MYC* cDNAs	[94]
Fanconi anemia	At present, 13 genes whose mutations cause Fanconi anemia are known	Skin fibroblasts and epidermal keratinocytes	Transduction with retroviruses carrying the *OCT4*, *SOX2*, *KLF4*, and *c-MYC* cDNAs. iPSCs from keratinocytes were produced without *c-MYC*	[87]

Induced pluripotent stem cells can become an alternative for ESCs in the area of clinical application of cell replacement therapy and screening for new pharmaceuticals. iPSCs closely resemble ESCs and, at the same time, can be produced in almost unlimited amounts from the differentiated cells of each patient. Despite the fact that the first iPSCs were produced relatively recently, work on directed iPSC differentiation and the production of patient–specific iPSCs is intensive, and progress in this field is obvious.

Dopamine and motor neurons were produced from human iPSCs by directed differentiation * in vitro * [71, 72]. These types of neurons are damaged in many inherited or acquired human diseases, such as spinal cord injury, Parkinson’s disease, spinal muscular atrophy, and amyotrophic lateral sclerosis. Some investigators have succeeded in producing various retinal cells from murine and human iPSCs [73–75]. Human iPSCs have been shown to be spontaneously differentiated * in vitro* into the cells of retinal pigment epithelium [76]. Another group of investigators has demonstrated that treating human and murine iPSCs with Wnt and Nodal antagonists in a suspended culture induces the appearance of markers of cell progenitors and pigment epithelium cells. Further treating the cells with retinoic acid and taurine activates the appearance of cells expressing photoreceptor markers [75].

Several research groups have produced functional cardiomyocytes (CMs) *in vitro * from murine and human iPSCs [77–81]. Cardiomyocytes produced from iPSC are very similar in characteristics (morphology, marker expression, electrophysiological features, and sensitivity to chemicals) to the CMs of cardiac muscle and to CMs produced from differentiated ESCs. Moreover, murine iPSCs, when injected, can repair muscle and endothelial cardiac tissues damaged by cardiac infarction [77].

Hepatocyte–like cell derivatives, dendritic cells, macrophages, insulin–producing cell clusters similar to the duodenal islets of Langerhans, and hematopoietic and endothelial cells are currently produced from murine and human iPSCs, in addition to the already–listed types of differentiated cells [82–85].

**Fig. 1:**
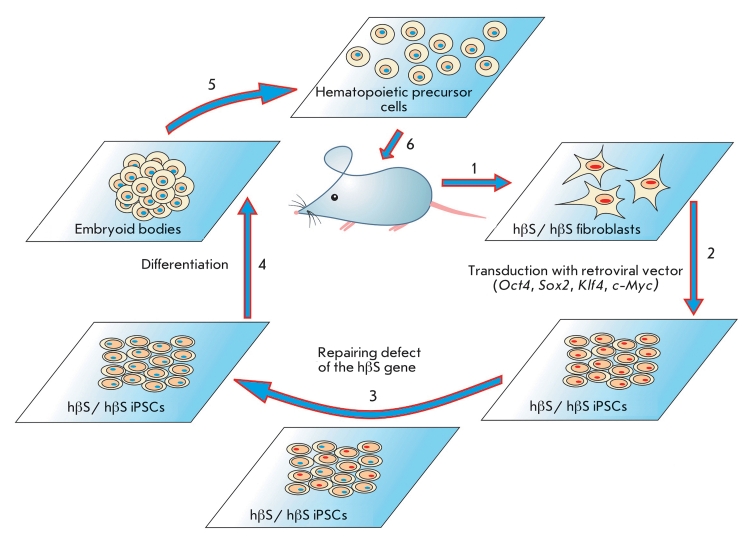
Design of an experiment on repairing the mutant phenotype in mice modeling sickle cell anemia development [2]. Fibroblasts isolated from the tail of a mouse (1) carrying a mutant allele of the gene encoding the human hemoglobin β-chain (hβs) were used for iPSC production (2). The mutation was then repaired in iPSCs by means of homological recombination (3) followed by cell differentiation via the embryoid body formation (4). The directed differentiation of the embryoid body cells led to hematopoietic precursor cells (5) that were subsequently introduced into a mouse exposed to ionizing radiation (6).

In addition to directed differentiation * in vitro * , investigators apply much effort at producing patient–specific iPSCs. The availability of pluripotent cells from individual patients makes it possible to study pathogenesis and carry out experiments on the therapy of inherited diseases, the development of which is associated with distinct cell types that are hard to obtain by biopsy: so the use of iPSCs provides almost an unlimited resource for these investigations. Recently, the possibility of treating diseases using iPSCs was successfully demonstrated, and the design of the experiment is presented in the figure. A mutant allele was substituted with a normal allele via homologous recombination in murine fibroblasts representing a model of human sickle cell anemia. iPSCs were produced from “repaired” fibroblasts and then differentiated into hematopoietic cell precursors. The hematopoietic precursors were then injected into a mouse from which the skin fibroblasts were initially isolated (Fig. 1). As a result, the initial pathological phenotype was substantially corrected [86]. A similar approach was applied to the fibroblasts and keratinocytes of a patient with Fanconi’s anemia. The normal allele of the mutant gene producing anemia was introduced into a somatic cell genome using a lentivirus, and then iPSCs were obtained from these cells. iPSCs carrying the normal allele were differentiated into hematopoietic cells maintaining a normal phenotype [87]. The use of lentiviruses is unambiguously impossible when producing cells to be introduced into the human body due to their oncogenic potential. However, new relatively safe methods of genome manipulation are currently being developed; for instance, the use of synthetic nucleases containing zinc finger domains allowing the effective correction of genetic defects * in vitro * [88].

The induced pluripotent stem cells are an excellent model for pathogenetic studies at the cell level and testing compounds possessing a possible therapeutic effect.

The induced pluripotent stem cells were produced from the fibroblasts of a patient with spinal muscular atrophy (SMA) (SMA–iPSCs). SMA is an autosomal recessive disease caused by a mutation in the * SMN1 * ( * survival motor neuron 1 * ) gene, which is manifested as the selective nonviability of lower α –motor neurons. Patients with this disorder usually die at the age of about two years. Existing experimental models of this disorder based on the use of flatworms, drosophila, and mice are not satisfactory. The available fibroblast lines from patients with SMA cannot provide the necessary data on the pathogenesis of this disorder either. It was shown that motor neurons produced from SMA–iPSCs can retain the features of SMA development, selective neuronal death, and the lack of * SMN1 * transcription. Moreover, the authors succeeded in elevating the SMN protein level and aggregation (encoded by the * SMN2 * gene, whose expression can compensate for the shortage in the SMN1 protein) in response to the treatment of motor neurons and astrocytes produced from SMA–iPSCs with valproate and torbomycin [89;]. iPSCs and their derivatives can serve as objects for pharmacological studies, as has been demonstrated on iPSCs from patients with familial dysautonomia (FDA) [90]. FDA is an inherited autosomal recessive disorder manifested as the degeneration of sensor and autonomous neurons. This is due to a mutation causing the tissue–specific splicing of the * IKBKAP * gene, resulting in a decrease in the level of the full–length IKAP protein. iPSCs were produced from fibroblasts of patients with FDA. They possessed all features of pluripotent cells. Neural derivatives produced from these cells had signs of FDA pathogenesis and low levels of the full–length * IKBKAP * transcript. The authors studied the effect of three substances, kinetin, epigallocatechin gallate, and tocotrienol, on the parameters associated with FDA pathogenesis. Only kinetin has been shown to induce an increase in the level of full–length * IKBKAP * transcript. Prolonged treatment with kinetin induces an increase in the level of neuronal differentiation and expression of peripheral neuronal markers.

Currently, a broad spectrum of iPSCs is produced from patients with various inherited pathologies and multifactorial disorders, such as Parkinson’s disease, Down syndrome, type 1 diabetes, Duchenne muscular dystrophy, β –talassemia, etc., which are often lethal and can scarcely be treated with routine therapy [51, 87, 89, 91–94]. The data on iPSCs produced by reprogramming somatic cells from patients with various pathologies are given in the Table 1.

One can confidently state that both iPSCs themselves and their derivatives are potent instruments applicable in biomedicine, cell replacement therapy, pharmacology, and toxicology. However, the safe application of iPSC–based technologies requires the use of methods of iPSCs production and their directed differentiation which minimize both the possibility of mutations in cell genomes under * in vitro * culturing and the probability of malignant transformation of the injected cells. The development of methods for human iPSC culturing without the use of animal cells (for instance, the feeder layer of murine fibroblasts) is necessary; they make a viral–origin pathogen transfer from animals to humans impossible. There is a need for the maximum standardization of conditions for cell culturing and differentiation.

## ABBREVIATIONS

ESC: embryonic stem cells
iPSCs: induced pluripotent stem cells
NSCs: neural stem cells
ASCs: adipose stem cells
PDFs: papillary dermal fibroblasts
CMs: cardiomyocytes
SMA: spinal muscular atrophy
SMA-iPSCs: iPCSs derived from fibroblasts of SMA patients
GFP: green fluorescent protein
LTR: long terminal repeat
